# Identification of *Enterocytozoon bieneusi* in goats and cattle in Thailand

**DOI:** 10.1186/s12917-019-2054-y

**Published:** 2019-08-28

**Authors:** Ruenruetai Udonsom, Rapeepun Prasertbun, Aongart Mahittikorn, Rachatawan Chiabchalard, Chantira Sutthikornchai, Attakorn Palasuwan, Supaluk Popruk

**Affiliations:** 1 Department of Protozoology, Faculty of Tropical Medicine, Mahidol University, Ratchawithi Road, Ratchathewi, Bangkok, 10400 Thailand; 2 Oxidation in Red Cell Disorders and Health Task Force, Department of Clinical Microscopy, Faculty of Allied Health Sciences, Chulalongkorn University, 154 Rama I Road, Bangkok, 10330 Thailand

**Keywords:** *Enterocytozoon bieneusi*, Goats, Cattle, Pigs, Zoonotic

## Abstract

**Background:**

*Enterocytozoon bieneusi* has been increasingly reported to infect domestic animals and humans, with human infections primarily reported as zoonotic in origin. The aim of the present study was to determine the presence and genotype of *E. bieneusi* in humans and domestic animals in central Thailand by testing stool samples of 200 apparently healthy humans, 73 goats, 60 cattle and 65 pigs using nested-PCR/ sequence analysis based on the ITS region of SSU rRNA genes.

**Results:**

*E. bieneusi* tested positive in 2 (1%) of the 200 stool samples collected from humans and 56 (28.3%) of the 198 stool samples collected from domestic animals. The highest prevalence of *E. bieneusi* was observed in pigs (39/65, 60%), followed by goats (14/73, 19.2%) and cattle (3/60, 5%). Seven novel *E. bieneusi* genotypes were identified, which were named GoatAYE1–4 and PigAYE1–3 and clustered in either zoonotic Group 1 or Group 2. Moreover, eleven previously described *E. bieneusi* genotypes were also identified (O, D, H, SX1, CHC8, CHG3, CS-10, SHZC1, LW1, WildBoar5, and EbpC). All novel genotypes exhibited zoonotic potential from a phylogenetic analysis of ITS region.

**Conclusion:**

Our data showed that the prevalence of *E. bieneusi* is low in apparently healthy individuals and higher in pigs than cattle and goats. This study provides baseline data useful for controlling and preventing *E. bieneusi* infection in farm communities, where pigs and goats appear to be the major reservoir of *E. bieneusi*. The results of our study support the view that *E. bieneusi* is a zoonotic pathogen that should be considered a potential public health threat.

## Background

*Enterocytozoon bieneusi*, a complex intestinal microsporidian pathogen with multiple genotypes, parasitises a variety of animals [1, 2] and causes infection both immunocompromised and immunocompetent humans including children, travellers and the elderly [3–5]. The clinical presentation of *E*. *bieneusi* infection ranges from being asymptomatic to presenting symptoms of dehydration, malabsorption and chronic diarrhoea [2]. In human, potential modes of *E*. *bieneusi* transmission are faecal-oral route and zoonotic [6–8]. Risk factors associated with *E*. *bieneusi* infection include impaired immunity, poor personal hygiene, deficient community sanitation and close contact with infected animals or humans [2].

Routine laboratory diagnosis of microsporidian infection involves staining and light microscopy. However, identification of this pathogen is challenging because of the small size of its spores [9], leading to underestimating of *E*. *bieneusi* prevalence. Polymerase chain reaction (PCR) is the most powerful, sensitive and specific technique for detecting and genotyping *E*. *bieneusi*. Based on PCR/sequence analysis of the internal transcribed spacer (ITS) region of small-subunit ribosomal RNA (SSU rRNA) genes, > 500 genotypes and 11 phylogenetic groups of *E*. *bieneusi* have been described in humans and animals [2, 10, 11]. *E*. *bieneusi* genotypes in Group 1 and Group 2 may be zoonotic genotypes responsible for most human infections [10, 12]. In animals, several studies indicated that pigs may be the major reservoir of *E*. *bieneusi* and a potential source of *E*. *bieneusi* infection in humans [8, 13–15]. Since there is limited information on the *E*. *bieneusi* genotypes in apparently healthy individuals in Thailand, most available information was studied in orphans and HIV-patients and previously studied target animals were only cats and pigs. We conducted this study to identify the prevalence and genotypes of *E*. *bieneusi* circulating among apparently healthy individuals and domestic animals especially, goats and cattle in central Thailand.

## Results

**Fig. 1:**
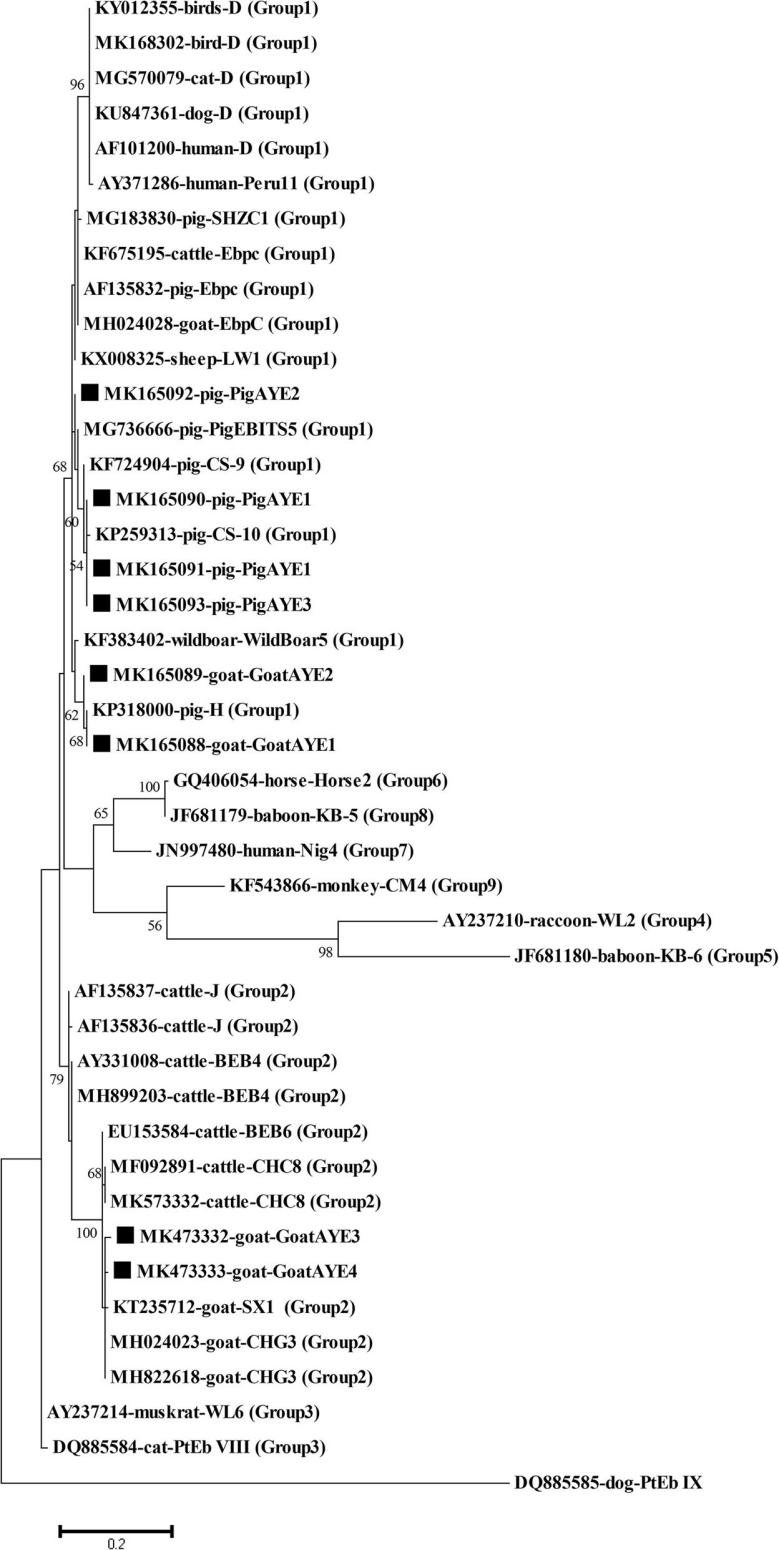
Phylogenetic tree of *E. bieneusi* isolates and reference for the internal transcribed spacer (ITS) region sequence of small-subunit ribosomal RNA (SSU rRNA) genes obtained from GenBank. The outgroup sequence was the dog-specific *E. bieneusi* genotype. Values on nodes represent bootstrap support using maximum likelihood methods. Symbol■ = novel genotypes identified in this study

The prevalence of *E. bieneusi* in animals and humans was 28.3 and 1%, respectively (Table 1). The highest prevalence of *E. bieneusi* occurs in pigs (60%), followed by goats (19.2%) and cattle (5%). Genotype analysis using the ITS region sequence in the 58 positive samples detected 18 different genotypes. These genotypes included 11 that are known [D, O, H, SX1, CHC8, CS-10, CHG3, LW1, EbpC, WildBoar5, and SHZC1] and seven genotypes that are novel [GoatAYE1–4 and PigAYE1–3]. The predominant genotypes of *E. bieneusi* identified in goats, pigs and cattle were CHC8/CHG3, EbpC and D, respectively. Our phylogenetic analysis revealed that the five novel genotypes were clustered in zoonotic Group1 (PigAYE1–3 and GoatAYE1–2), whereas the others were in zoonotic Group2 (GoatAYE3–4) (Fig. 1).

**Table 1:** Prevalence and genotypes of *Enterocytozoon bieneusi* in Ayutthaya Province, Thailand

Host	Prevalence (%)	Genotype (synonyms)/ Positive count (GenBank accession no.)
Humans	2/200 (1)	O/ 1 (KU245704)
		D (WL8, Peru9, PigEBITS9, PtEb VI, CEbc)/ 1 (MK168302)
Animals		
Goats	14/73 (19.2)	GoatAYE1^a^/ 1
		GoatAYE2^a^/ 1
		GoatAYE3^a^/1
		GoatAYE4^a^/1
		H (PEbC) / 1 (KP318000)
		SX1/ 1 (KT235712)
		CHC8/ 4 (MK573332)
		CHG3/4 (MH822618)
Pigs	39/65 (60)	CS-10/ 1 (KP259313)
		SHZC1/1 (MG183830)
		PigAYE1^a^/ 2
		PigAYE2^a^/ 1
		PigAYE3^a^/ 1
		H (PEbC)/ 2 (KP318000)
		LW1 (Henan-I) / 6 (KX008325)
		WildBoar5/ 9 (KF383402)
		EbpC (E,Peru4,WL13,WL17)/ 16 (MH024028)
Cattle	3/60 (5)	D (WL8, Peru9, PigEBITS9, PtEb VI, CEbc) / 3 (MK168302)
Total	56/198 (28.3)	
Total	58/398 (14.6)	

^a^Novel genotypes found in this study

Table 2 shows nucleotide differences between the seven novel genotypes and previously identified genotypes. The novel genotypes GoatAYE1 and GoatAYE2 showed one nucleotide difference in the ITS region relative to genotype H at position 245 C → T and position 126 A → G, respectively. The novel genotype GoatAYE3 showed two nucleotide differences in the ITS region relative to genotype CHC8 (position 95 T → C and 163 G → A), whereas GoatAYE4 showed one nucleotide difference from genotype CHG3 (position 134 G → A). Moreover, the novel genotype PigAYE1 showed one nucleotide difference from genotype CS-10 (position 21 G → A), while PigAYE3 showed two nucleotides difference relative to CS-10 (position 21 G → A and position 246 A → C). In addition, PigAYE2 showed one nucleotide difference to genotype PigEBITS5 (position 131 T → C).

**Table 2:** Variations in ITS region sequences of SSU rRNA genes of *Enterocytozoon bieneusi* isolates identified in this study and comparison with three known genotypes

Genotypes (no.)		Nucleotide at position								GenBank accession no.
		21	95	126	131	134	163	245	246	
Novel	GoatAYE1	–	–	–	–	–	–	T	–	MK165088
	GoatAYE2	–	–	G	–	–	–	–	–	MK165089
Known	H	–	–	A	–	–	–	C	–	KP318000
Novel	GoatAYE3	–	C	–	–	–	A	–	–	MK473332
Known	CHC8	–	T	–	–	–	G	–	–	MK573332
Novel	GoatAYE4	–	–	–	–	A	–	–	–	MK473333
Known	CHG3	–	–	–	–	G	–	–	–	MH822618
Novel	PigAYE1	A	–	–	–	–	–	–	–	MK165090/ MK165091
	PigAYE3	A	–	–	–	–	–	–	C	MK165093
Known	CS-10	G	–	–	–	–	–	–	A	KP259313
Novel	PigAYE2	–	–	–	C	–	–	–	–	MK165092
Known	PigEBITS5	–	–	–	T	–	–	–	–	MG736666

## Discussion

In this study, the overall prevalence of *E. bieneusi* in humans and animals was 14.6%. The highest prevalence of *E. bieneusi* in animals occurs in pigs (60%), followed by goats (19.2%) and cattle (5%), whereas the prevalence of *E. bieneusi* in humans was detected in only 1%. Other studies in Thailand found that the prevalence of *E. bieneusi* in humans ranged from 1.3–27.27% and varied from country to country [8, 16–20]. Divergences in the prevalence of infection might due to differences in the immunity of hosts when stool samples were collected, personal hygiene, cultural norms, and detection methods. The prevalence of *E. bieneusi* infection in pigs observed in our study was higher than prevalence reported by other studies in Thailand and elsewhere, such as in China and Central Europe (5.4–28.1%) [8, 17, 21, 22], but similar to a study reported by Fiuza et al. in pigs in Brazil (59.3%) [14]. There are only a few studies reported a higher prevalence of *E. bieneusi* in pigs than our study (83.2–94%) [23, 24]. We also found that there was a higher prevalence of *E. bieneusi* in pigs aged 2–4 months than other ages, which is consistent with studies on pigs from Brazil, China, and Thailand [14, 17, 25, 26]. The higher prevalence among piglets may due to lower immunity and higher stress [27]. Moreover, cages with a high density of pigs may cause more opportunities for transmitting *E. bieneusi* infection among animals than less densely-packed cages. To the best of our knowledge, this is the first study to report the prevalence of *E. bieneusi* in cattle (5%) and goats (19.2%) in Thailand. The low prevalence of *E. bieneusi* in cattle found in Thailand contradicts the results of studies conducted in Argentina, Brazil, China, and South Korea [28–32]. The prevalence of *E. bieneusi* in goats was also lower than the prevalence rates found in previous studies from China and Egypt (20.5–28.8%) [33–36], except for the studies by Li et al. and Lores et al. (4.1–14.2%) [37, 38]. These diversities between studies in prevalence among animals might be related to the age and/or immunity of sampled animals, as well as to geographical differences. In our study, most of the tested cattle and goats were older than six months, so the experimental animals may have already developed immunity against *E. bieneusi*.

In this study, we detected 11 previously described and seven novel genotypes of *E. bieneusi* in human and animal samples. In Thailand, previous studies have determined that genotype D is commonly observed in both HIV-positive patients, healthy individuals and animals, such as in Indian peafowl and calf [8, 19, 28, 39–42]. This genotype was found from all *E. bieneusi*-positive cattle in this study.

Genotype O has been observed in humans, pigs, cattle and dogs in previous studies conducted in various countries worldwide [8, 14, 19, 24, 30, 43]. Nine of 18 *E. bieneusi* genotypes identified in our study occurred in pigs with the predominant genotype consisting of EbpC, followed by LW1 and H. In Thailand, genotypes EbpC and H have been reported in humans and pigs [8, 17, 19], which is consistent with previous studies conducted in other countries [14, 24, 44, 45]. Genotype EbpC has also been identified in cattle, dogs, goats, and monkeys [28, 43, 46, 47], suggesting that this genotype is not host-specific. Although genotype LW1 was reported for the first time from pigs in Thailand, this genotype has been previously identified in humans, pigs and wild boars in Australia and China [22, 24, 25, 45]. Surprisingly, five of 39 *E. bieneusi*-infected pigs we genotyped as WildBoar5, a genotype that has also been identified from wild boars in Central Europe [22]. Due to the fact that pig and wild boar belong to the same species, they are likely parasitised by the same species [22]. Moreover, genotypes CS-10 and SHZC1 in pigs, genotypes SX1, CHC8 and CHG3 in goats have also been identified and consistent with previous studies [26, 27], except that CHC8 has been previously identified in cattle [29, 34, 47]. Phylogenetic analyses provided further information that the five novel genotypes were clustered in zoonotic Group1 (PigAYE1–3 and GoatAYE1–2) and the others were grouped in zoonotic Group 2 (GoatAYE3–4), which implies that they have tremendous potential to be zoonotic. Apparently, genetic diversity definitively exists in this pathogen and zoonotic potential of these novel genotypes should be characterised further in future studies.

## Conclusions

Our results revealed a high prevalence of *E. bieneusi* in pigs (60%) and identified a high number of zoonotic genotypes, while goats and cattle were also carriers of *E. bieneusi.* Therefore, it is important to control *E. bieneusi*-infected animals on farms by properly managing animal waste. To better understand the molecular epidemiology and zoonotic potential of *E. bieneusi*, it will be necessary to increase sample size, examine a wider variety of animal species and expand the survey area.

## Methods

### Specimen collection

**Fig. 2:**
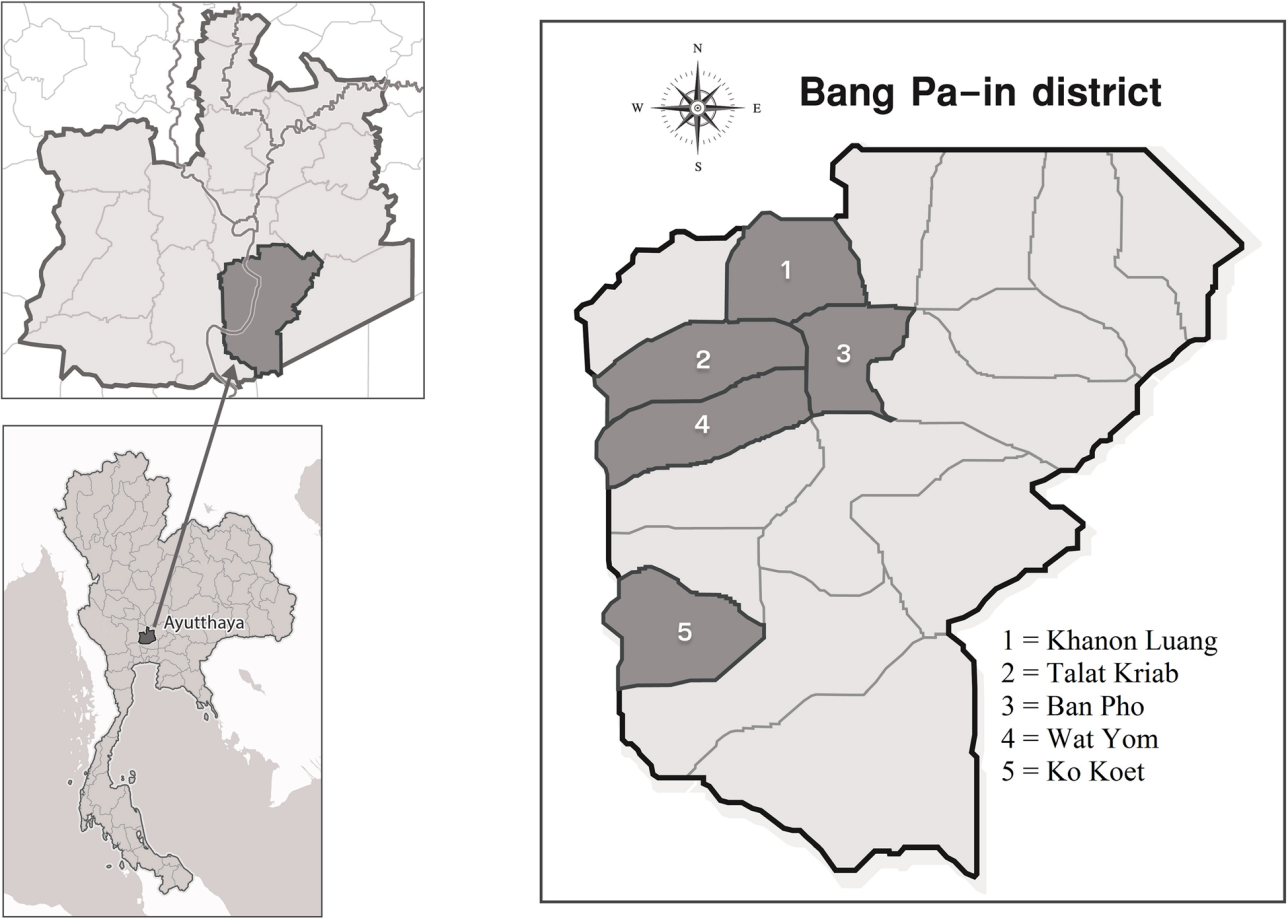
Map of study areas in Bang Pa-In District, Ayutthaya Province, central Thailand (Design by miss Chompunuch Sangpan (our co-worker in Faculty of Tropical Medicine))

A cross-sectional study of *E. bieneusi* infection was conducted from five Thai sub-districts (Khanon Luang, Talat Kriap, Ban Pho, Wat Yom, and Ko Koet) in the Bang Pa-In District Ayutthaya Province**,** central Thailand. In total, 200 apparently healthy individual stool samples and 198 stool samples from domestic animals, including 73 goats (Farms 1–3), 60 cattle (Farms 4–6) and 65 pigs (Farms 7–9) and human participants aged from 1 to 80 years (Fig. 2). No participants complained about any gastrointestinal symptoms, such as abdominal pain or diarrhoea throughout the experiment. All nine farms participated in our study were small and private farms. Ages of goats on Farms 1–3 ranged between 4 and 12 months, ages of cattle on Farms 4–6 were more than 6 months, while ages of pigs on Farms 7, 8 and 9 ranged between 2 and 5 months, 3–6 months and 3–8 months, respectively. Types of animals were selected based primarily on their proximity to humans in experimental areas. Fresh stool samples were collected either directly from animals’ rectum; each animal was securely detained in an individual cage, or immediately after defecation (each sample was separately taken from the middle of the stool with a clean plastic spoon).

All stool samples were safely stored under cool conditions during transportation to the Department of Protozoology, Faculty of Tropical Medicine, Mahidol University and preserved at -20 °C before extracting DNA.

### DNA extraction and nested PCR

DNA was extracted from stool samples using a commercially available DNA extraction kit (PSP^Ⓡ^ Spin Stool Kit, Stratec Molecular, Berlin, Germany) following the manufacturer’s instructions. We amplified a fragment of the ITS region of SSU rRNA genes from the extracted DNA using nested PCR. The amplicons were about 390 base pairs (bps) in length [13]. The outer primer set was EBITS3 (5′- GGT CAT AGG GAT GAA GAG − 3′) and EBITS4 (5′- TTC GAG TTC TTT CGC GCT C-3′), whereas the inner primer set was EBITS1 (5′- GCT CTG AAT ATC TAT GGC T-3′) and EBITS2.4 (5′- ATC GCC GAC GGA TCA AGT G-3′). The thermal cycling conditions were maintained as follows: initial denaturation at 94 °C for 3 min, 35 cycles of denaturation at 94 °C for 30 s, annealing at 55 °C for 30 s, extension at 72 °C for 40 s, followed by final extension at 72 °C for 10 min. The final PCR products (390 bps) were separated on 2% agarose gel and visualised under a UV transilluminator.

### Sequencing and phylogenetic analysis

All positive PCR products from the second PCR reaction were purified before sequencing with an ABI 3730xl DNA analyser using the BigDye Terminator Cycle Sequencing kit (Applied Biosystems). All sequences of *E. bieneusi*-positive samples were checked for similarity with previously published sequences stored in the GenBank database using BLAST (https://blast.ncbi.nlm.nih.gov/Blast.cgi). The nucleotide sequences of novel *E. bieneusi* isolates were submitted to GenBank under accession numbers MK165088–MK165093 and MK473332–MK473333.

The sequences of *E. bieneusi*-positive samples and an outgroup sequence (GenBank accession no. DQ885585) were aligned using Clustal W and phylogenetic analysis was performed with MEGA version 6 software (http://www.megasoftware.net) [48]. The Tamura-Nei model was used to account for the evolution of the DNA sequences and constructed a phylogenetic tree using maximum likelihood methods with 1000 bootstrap replicates.

### Statistical analysis

Descriptive analyses (percentages) were used to describe the prevalence of positive stool samples and the distributions of *E. bieneusi* genotypes throughout the study region.

## Data Availability

The data sets used and/or analysed during the present study are available from the corresponding author by reasonable request.

## Abbreviations

bps: base pairs
ITS: Internal transcribed spacer
PCR: Polymerase chain reaction
SSU rRNA: Small-subunit ribosomal RNA
